# *In silico* search for modifier genes associated with pancreatic and liver disease in Cystic Fibrosis

**DOI:** 10.1371/journal.pone.0173822

**Published:** 2017-03-24

**Authors:** Pascal Trouvé, Emmanuelle Génin, Claude Férec

**Affiliations:** 1 Inserm, UMR1078, Brest, France; 2 Université de Bretagne Occidentale, Faculté de Médecine et des sciences de la santé, Brest, France; 3 C.H.U. Brest, Hôpital Morvan, Laboratoire de Génétique Moléculaire, Brest, France; 4 Etablissement Français du Sang - Bretagne, Brest, France; Centro Nacional de Investigaciones Oncologicas, SPAIN

## Abstract

Cystic Fibrosis is the most common lethal autosomal recessive disorder in the white population, affecting among other organs, the lung, the pancreas and the liver. Whereas Cystic Fibrosis is a monogenic disease, many studies reveal a very complex relationship between genotype and clinical phenotype. Indeed, the broad phenotypic spectrum observed in Cystic Fibrosis is far from being explained by obvious genotype-phenotype correlations and it is admitted that Cystic Fibrosis disease is the result of multiple factors, including effects of the environment as well as modifier genes. Our objective was to highlight new modifier genes with potential implications in the lung, pancreatic and liver outcomes of the disease. For this purpose we performed a system biology approach which combined, database mining, literature mining, gene expression study and network analysis as well as pathway enrichment analysis and protein-protein interactions. We found that IFI16, CCNE2 and IGFBP2 are potential modifiers in the altered lung function in Cystic Fibrosis. We also found that EPHX1, HLA-DQA1, HLA-DQB1, DSP and SLC33A1, GPNMB, NCF2, RASGRP1, LGALS3 and PTPN13, are potential modifiers in pancreas and liver, respectively. Associated pathways indicate that immune system is likely involved and that Ubiquitin C is probably a central node, linking Cystic Fibrosis to liver and pancreatic disease. We highlight here new modifier genes with potential implications in Cystic Fibrosis. Nevertheless, our *in silico* analysis requires functional analysis to give our results a physiological relevance.

## Introduction

Cystic Fibrosis (CF) is the most common lethal autosomal recessive disorder in the white population. Its incidence is one in 2,500 with a carrier frequency of one in 25. The Cystic Fibrosis Transmembrane Conductance Regulator (CFTR) gene, causing CF, was identified in 1989 [1] and located on chromosome 7q31.2, spanning a transcription unit of about 216.7 kb with 27 exons [2]. It encodes a transmembrane protein (CFTR, 1,480 amino acids) which is an ATP-binding cassette transporter functioning as a chloride (Cl^-^) channel [1–6]. The CFTR channel’s opening requires phosphorylation by cAMP-dependent protein kinases [6–8] and hydrolyzable MgATP [8]. The CFTR protein is located at the apical membrane of polarized epithelial cells in diverse tissues including the lungs, sweat ducts, pancreas, gastrointestinal tract, and vas deferens [9, 10].

There are currently 2,009 mutations listed in the CFTR mutation database (http://www.genet.sickkids.on.ca/cftr/app). The most common one, F508del-CFTR (a 3 bp deletion in exon 10 causing loss of the amino acid phenylalanine at position 508), encodes a cAMP-regulated Cl^-^ channel that is retained in the endoplasmic reticulum (ER) during translation and folding and is targeted to the early proteasomal degradation [11]. Besides an altered folding, the F508del-CFTR protein exhibits an altered function with an open time period of approximately 0.1 to 0.3 seconds [12, 13].

The phenotype due to a given mutation depends on its interaction with the second mutated CFTR allele and largely on disease modifiers. Whereas CF is a monogenic disease, there is a very complex relationship between genotype and phenotype [14]. A good genotype-phenotype correlation is observed in the pancreas. Severe and mild mutations are associated with pancreatic insufficiency (PI) and pancreatic sufficiency, respectively [15]. Although CFTR genotype is clearly predictive of PI [16], the genotype-phenotype link is unclear regarding lung and liver diseases. Environmental factors such as infection, nutritional status or socioeconomic status may influence in the pulmonary phenotype in CF. Nevertheless, they can’t explain the degree of variability observed in patients exhibiting a same CFTR genotypes. This is shown by studies in CF twins indicating that other genetic factors may explain the observed variability [17, 18]. Modifier genes may play a significant role in determining the severity of CF. Furthermore, for a single mutation such as F508del, the pulmonary status may range from insignificant to severe [19, 20]. It is therefore obvious that, whereas classic genotype-phenotype studies in CF are important, they are not sufficient and have to be complemented by the search for environmental effects on the phenotype of patients, to increase the basic knowledge of the disease and to develop new therapeutic approaches.

The influence of genetic modulators on CF [21] shows the importance to search for potential candidate gene acting upon CF phenotype. For a given CF genotype, modifier genes may influence the phenotype via the same or parallel pathways [22], leading to complex studies. Therefore, in many works, the candidate gene approach is used. Candidate genes are search in the interactome of the disease gene or in a pathway that is indirectly involved in the course of the disease. Some genome-wide association meta-analysis also identified modifier loci of lung disease severity [23]. In the present work, a different methodology was used.

To search for genetic factors that may play a role in CF, we used a system biology approach. This approach combined database and literature mining, gene expression, and network analysis. Pathway enrichment analysis and protein-protein interactions (PPI) were also searched, to give our results a physiological relevance. This approach allowed us to examine functional relationships between reported genes, and led us to identify novel genes and enriched pathways that may play a role in CF, regarding its lung, pancreatic and liver affections. We found that genes involved in immunity including Ubiquitin C are likely modifiers.

## Materials and methods

### Data sources and gene selection from databases

The gene selection was performed using a previously used methodology [24].

Genes were first identified utilizing the Online Mendelian Inheritance in Man (OMIM) database (http://www.omim.org/) because it is considered to be the best curated resource for genotype-phenotype relationships [25, 26]. In OMIM, we used the following key words: CF, CF associated genes, PI and Liver Disease. CF and CF associated genes were used to retrieve genes directly involved in CF as well as genes potentially implicated in the disease.

The Comparative Toxicogenomics Database (CTD, http://ctdbase.org/, [27]) was also used. It curates relationships between genes and human diseases in a unique fashion which integrates gene/protein-disease relationships. In CTD, CF, Pancreatic diseases and Liver Disease key words were used. In CTD, disease-gene associations are reported as curated or inferred. We selected curated associations due to a higher confidence than inferred associations. Solely the genes that may be biomarkers of a disease or play a role in the etiology of a disease were selected.

Lastly, the Human Genome Epidemiology encyclopedia (HuGE Navigator, http://www.hugenavigator.net/HuGENavigator/startPagePubLit.do, [28]) which mines the scientific literature on human gene-disease associations was used. It is a database of population-based epidemiologic studies of human genes [29]. In HuGE Navigator we used the CF, PI and Liver Disease key words. The genes with a genetic association above 5 were selected (score formula is described in [30]).

Different key words were used among databases because some of them did not permit to retrieve any result.

Venn diagrams were drawn to find common genes from these databases.

### Comparison with published gene lists from bibliography

Differentially expressed genes in CF were retrieved from works published in PubMed (http://www.ncbi.nlm.nih.gov/pubmed). Up- and down-regulated genes were retrieved from a paper [31] in which 4 independent studies [32–35] and a re-analyzed list of genes [36] were compared. We used here a list of 75 up-regulated and 114 down-regulated genes which are shared by these studies and having the same direction of expression in at least two studies [31–37]. These genes are given in Table 1.

**Table 1:** Retrieved differentially expressed genes in CF (adapted from [31]). The lower part of the table shows genes shared between two or more studies when all six studies are combined. Common genes in at least three studies are underlined.

Origin of the gene lists (References)	Regulated genes shared with [31]
[33]	**UP:** ACAA2, CDKN2B
	**DOWN:** IGFBP2
[34]	**UP:** C9orf3, KRT14
	**DOWN:** CYP24A1, HLA-DQA1, SAA4
[32]	**UP:** CAV1, CCNE2
	**DOWN:** CLGN, ENO2, EPB41L3, GPX3, TIMP4
[36]	**UP:** BCL2A1, G0S2, IL1B, MMP1, RGS2
	**DOWN:** CKB, CRIP1, CYP24A1, DNALI1, FHL1, GSTT1, IGFBP2
[37]	**UP:** BCL2A1, G0S2, IL1B, IL1R2, LCP2, NDRG1, RGS2, RNF149, TCN1
	**DOWN:** PROS1, SCGB1A1, SPAG8
**All shared genes in the 6 studies** [31–35, 37]	
**UP** (n = 75): ACAA2, AGL, AKR1C1, ANXA8L2, BCHE, BLOC1S1, BTBD3, C9ORF3, CAPG, CAV1, CCL20, CCNE2, CD24, CD83, CDKN2B, CLGN, CSF3, DDX3Y, ELK3, FOLR1, FOLR3, FOXG1, GCA, GFPT2, HCLS1, HIST1H1C, HMOX1, HPCAL1, HSPB11, IFIT1, IFIT3, IFITM1, IL1R2, IL7R, ISG15, KCTD12, KRT14, KRT81, LCP2, LITAF, LYPD1, MLF1, MMP1, MRPL28, MX2, NCF1, NDRG1, NET1, PLAU, PLAUR, PLTP, PRSS3, PSG9, PTPN13, RAC2, RAGE, RNF149, RPA3, SEMA3B, SERPINA3, SERPINF1, SLITRK5, SOD2, SULT1A3, TCF15, TCIRG1, TCN1, TXNIP, BCL2A1, G0S2, IL1B, NCF2, PLAT, PTGS2, RGS2.	
**DOWN** (n = 114): ACTA2, ADAR, ALDH1A1, ANKRD1, ASNS, ASS1, BEX4, BST2, BTG1, C5ORF13, CALD1, CAP1, CCL20, CD164, CFB, CGREF1, CKB, CLGN, COL8A1, COL9A3, CRIP1, CSTA, CXCR4, CYP51A1, DDB2, DDIT4, DNALI1, DSP, DSTN, DYNLT1, EDNRA, EFEMP1, EIF4A2, ENO2, EPB41L3, EPS8, F3, FBLN5, FCGBP, FHL1, GABRP, GCH1, GINS1, GPNMB, GPR1, GPX3, GSTT1, GZMB, HCP5, HES1, HLA-B, HLA-DQA1, HLA-DRA, HLA-DRB1, HLA-F, HLA-G, HMGCS1, HSPB1, HTRA1, ID1, IFI16, IFITM1, IFRD1, IGFBP7, IL32, KCNN4, KIT, KLRK1, KRT15, LCN2, LGALS3BP, LOX, MGAM, MSC, NID2, NPR3, NTS, PGD, PNMA2, PPP1R3C, PROS1, RASGRP1, RND3, RUNX3, SAA4, SC5DL, SCGB1A1, SERPINB3, SERPINB4, SGK1, SLC2A3P1, SNAPC1, SPAG8, STAC, STAT4, TES, TFPI, THBD, TIMP4, TPBG, TRIB2, TWIST1, VDAC1, ZNF643, CTSC, CYP24A1, IFI27, IGFBP2, IGFBP3, NAMPT, PRSS23, SEL1L3, TMSB4X, TRIM22	

To search for up- and down-regulated genes in Liver Disease, we focused on gene expression in Human non alcoholic steato-hepatitis (NASH, [38–41]). The rationale that liver disease in NASH and liver of patients with CF share common pathways is based on the fact that steatosis is one of the most common hepatic affection in CF (prevalence of 23–75%). It is observed in 70% of children with liver disease, independently of their nutritional status. Together with a uniform hyperechogenicity of their liver, some pseudomasses may be seen by ultrasound and are due to lobulated fatty structures. In about 60% of the cases, the steatosis is associated with hepatic enzymes elevation (aminotransferases). Because this is observed in children, it is likely not due to alcohol consumption. Therefore, beside the many liver affections observed in livers from CF patients, they present many features of NASH (for review: [42]). Liver Disease key word was not used because it did not permit to retrieve studies with gene analysis. In a first study [38], 16 genes were differentially expressed in subjects with NASH when compared with healthy controls. 12 and 4 genes were significantly under- and 4 over-expressed in NASH, respectively. In a second study [39] comparing NASH samples versus controls, 14 genes were found with more than a two-fold difference. In the third study [40], comparison of non-obese controls patients with NASH exhibited 34 differentially expressed genes (> two fold). We noticed that the expression of 19 genes among 34, were not different in the obese and non-obese controls, showing that the second study using obese controls could be used here. In a last study [41], 9724 genes were differentially expressed in the normal liver versus NASH, (21,619 genes were unchanged).

To search for disease-specific genes in Chronic Pancreatitis, a study showing 152 and 34 genes with increased and decreased expression, respectively, was used [43]. Full name and OMIM identification for these 186 genes was searched. We used another study from which we extracted specific genes in Chronic Pancreatitis [44].

In our study, some genes were withdrawn because the corresponding record was deleted by NCBI or because the term was not found in Gene records. Pseudogenes and non human genes (artifacts) were also withdrawn.

### Expression analysis

Datasets restricted to humans, were collected from the Gene Expression Omnibus (GEO, [45]) and GEO2R was used for the analysis [46]. For this purpose, experimental groups were re-assigned. The following datasets were passed through the GEO2R online application: GSE40445 (Gene expression in CF-vs-non CF airway epithelial cells from nasal brushing), GSE48452 (Gene expression in control liver-vs-NASH) and GSE44314 (Gene expression in type 1 diabetes-vs-healthy controls). Type 1 diabetes was used because no PI dataset was found. Samples were assigned to the case or control groups. The GEO2R uses the limma (Linear Models for Microarray Data) package from Bioconductor and was designed to analyze complex experiments involving comparisons between many RNA targets simultaneously, with the idea to fit to a linear model to the expression data for each gene. Whereas limma provides several p-value options, we applied the adjustment to the p-values also called multiple-testing corrections, to correct the occurrence of false positive results. The *Benjamini & Hochberg false discovery rate* method, commonly used to adjust microarray data, was selected. After GEO2R dataset analysis, genes of interest were selected by using the criteria of a Bonferroni-corrected p-value ≤ 0.05. According to the annotation files, probes that were not corresponding to any genes and genes which were present several times within a single study were eliminated. Differentially expressed transcripts between normal and pathologic were selected and common genes between GSE40445, GSE48452 and GSE44314 were searched by drawing a Venn diagram (not shown).

### Analysis of enrichment of KEGG biochemical pathways in CTD

Our target genes were mapped to biological process pathways using CTD (http://ctd.mdibl.org). Biocurators at CTD manually curated gene-disease relationships from the literature as well as pathways. The core data are integrated to construct pathway networks. The VennViewer function, which permits to compare associated datasets, was used for the genes that were found to be potential modifiers in CF, Liver Disease and Chronic Pancreatitis. The Pathway associations function was selected to compare annotated enriched pathways, as well as pathways for disease for these genes. Associated data for interacting genes and diseases, inferred KEGG and REACTOME pathways, or enriched GO terms were downloaded. Annotated pathways for genes are associations established by KEGG and REACTOME curation. The significance of a given pathway is reported as a p-value.

### Gene-disease association search

Gene-disease associations were search in CTD in which associations are extracted from the published literature or are derived from the OMIM database using the mim2gene file from the NCBI Gene database. Curated associations were retrieved according to their Bonferroni-corrected p-value < 0.01.

### PPI networks design

PPI networks were obtained by STRING 10.0 (Search Tool for the Retrieval of Interacting Genes and Proteins, http://string-db.org/). STRING is a global resource for searching connections (edges) between genes or proteins (nodes) currently covering 9,643,763 proteins. Each edge has a confidence value between 0 and 1, lowest and highest confidence, respectively. The gene’s names were used as an initial input and experimental data sets were selected to display interactive networks. The parameters were defined as follows: all PPI prediction methods were enabled, except for High-throughput Lab Experiments; maximum of 5 interactions by node; cut-off criterion of combined score ≥ 0.7 (interactions at high confidence or better); Homo sapiens.

## Results and discussion

### Study design

**Fig 1:**
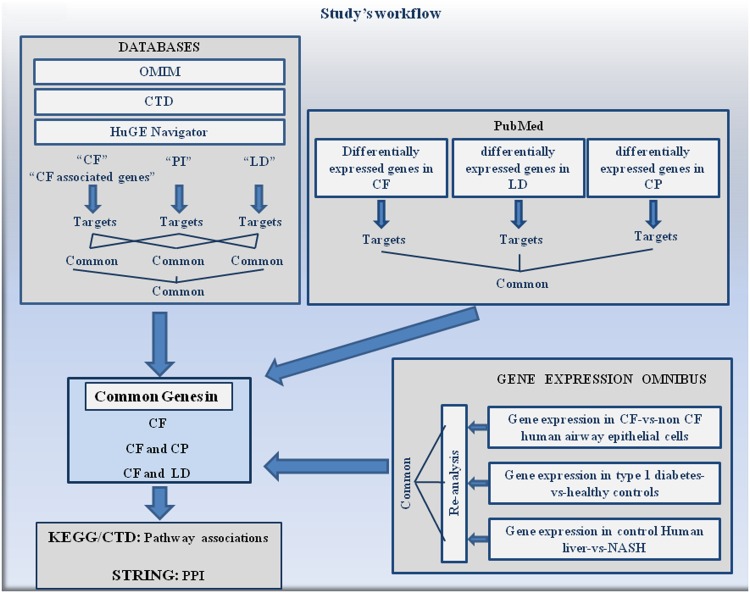
Workflow of the study. Common genes in CF, PI and Liver Disease were retrieved from OMIM, CTD and HuGE. Candidate genes were also retrieved from published works (PubMed) and datasets were re-analyzed in GEO. Potential modifier genes from the 3 different origins were compared and analyzed to search for pathways and protein-protein interactions.

Fig 1 depicts the workflow of our study. We retrieved genes from OMIM, CTD and HuGE. Common genes were searched in CF, pancreatic disease and Liver Disease. Differentially expressed genes in CF were also retrieved from published results in PubMed. Finally, Datasets were collected from the GEO and re-analyzed. Because the CF, *Chronic Pancreatitis* and Liver Disease keywords did not give any result, type 1 diabetes-vs-healthy control and control liver-vs-NASH were used in GEO. Candidate genes from these databases were compared and analyzed to search for potential common genes and pathways. PPI were further searched to give our results a physiological relevance.

### Gene selection from databases

**Fig 2:**
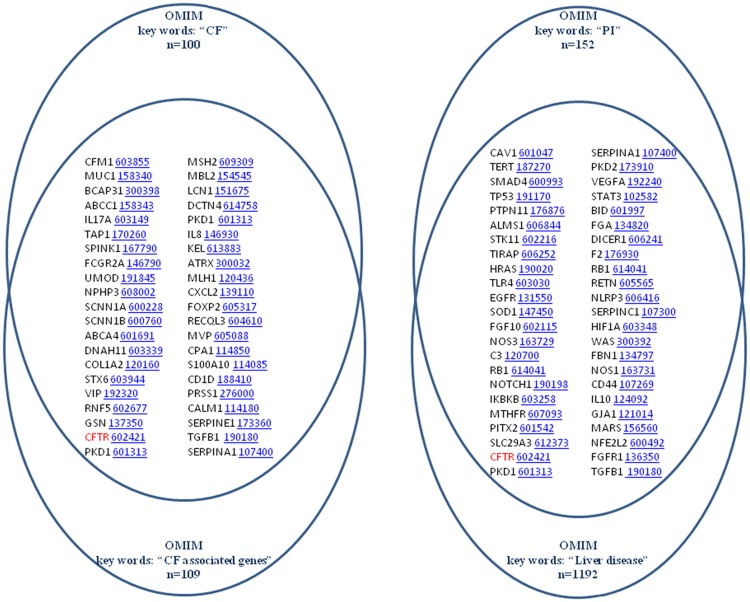
Venn diagrams of the OMIM search. 42 different genes were found to be common in CF and CF associated genes (left). 46 genes were shared by PI and Liver Disease (right).

**Fig 3:**
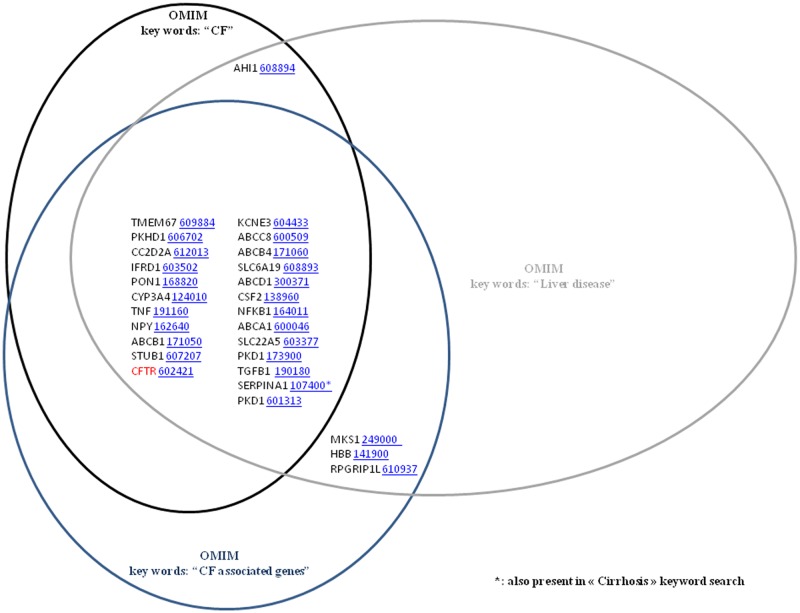
Venn diagrams showing the common genes in CF, CF associated genes and Liver Disease (OMIM). 24 genes were found in common in CF, CF associated genes and Liver Disease.

OMIM is a comprehensive, daily-updated human phenotype database, containing more than 12,000 genes of all human genetic diseases. The searches in OMIM using CF, CF associated genes, PI and Liver Disease key words led to 100, 109, 152, 1192 genes, respectively (Fig 2). The intersection of CF and CF associated genes showed 42 different genes and 46 genes were shared by PI and Liver Disease. The 6 genes shared by CF, CF associated genes and PI keywords were S100A8/S100A9 (OMIM Accession ID: 123885), CFTR (OMIM Accession ID: 602421), SCNN1G (OMIM Accession ID: 600761), TGFB1 (OMIM Accession ID: 190180), SERPINA1 (OMIM Accession ID: 107400) and PKD1 (OMIM Accession ID: 601313). In the intersection of CF plus CF associated genes with Liver Disease key words, we found 24 genes (Fig 3). Finally, the number of genes in the intersection of the pooled CF and CF associated genes search and the pooled PI and Liver Disease search was only 4. These genes were CFTR (OMIM Accession ID: 602421), TGFB1 (OMIM Accession ID: 190180), SERPINA1 (OMIM Accession ID: 107400) and PKD1 (OMIM Accession ID: 601313).

**Fig 4:**
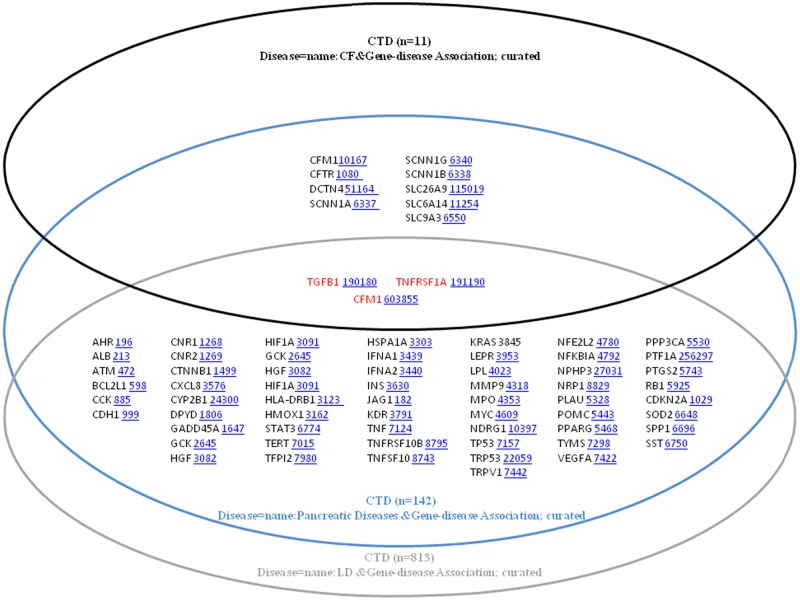
Venn diagrams of the CTD database search. The 3 shared genes in CF, Pancreatic diseases and Liver Disease were TGFB1, TNFRSF1A and CFM1.

Using the CTD database, 11, 142 and 813 genes were retrieved using the CF, Pancreatic diseases and Liver Disease key words, respectively. Fig 4 presents a Venn diagram showing the shared genes between these key words. Only 3 genes were found to be shared among the 3 used key words: TGFB1 (NCBI Accession ID: 7040), TNFRSF1A (NCBI Accession ID: 7132), CFM1 (NCBI Accession ID: 10167). Surprisingly, among some differences with the OMIM search, the CFTR gene was not found to be present in the list of genes retrieved using the Liver Disease key word in CTD.

**Fig 5:**
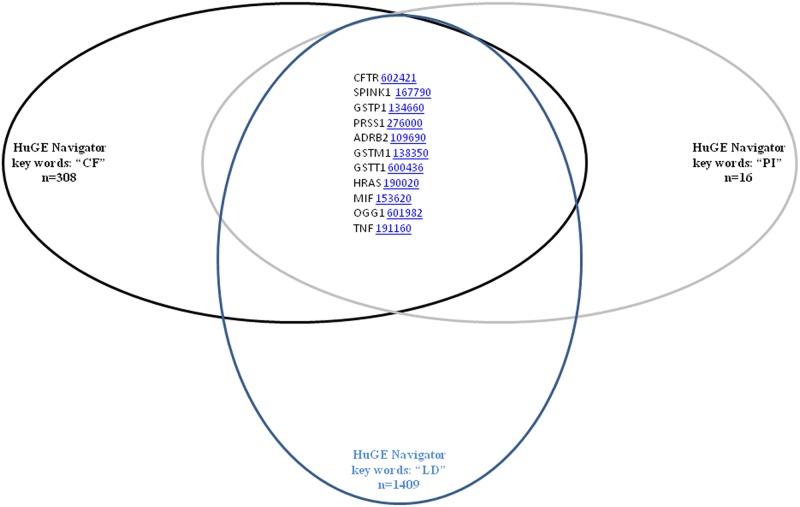
Venn diagrams of the HuGE Navigator database search. Common genes in PI, CF and Liver Disease were CFTR, SPINK1, GSTP1, PRSS1, ADRB2, GSTM1, GSTT1, HRAS, MIF, OGG1 and TNF. (score > 5).

HuGE Navigator database is an integrated, searchable, Web-based knowledge base which mines the scientific literature on human genetic associations and human genome epidemiology [29, 30]. In HuGE Navigator, common genes (score > 5) in PI, CF and Liver Disease were CFTR, SPINK1, GSTP1, PRSS1, ADRB2, GSTM1, GSTT1, HRAS, MIF, OGG1 and TNF. The corresponding OMIM Accession IDs are given in Fig 5.

### Differential gene expressions retrieved from bibliographic analysis

Differentially expressed genes in CF were retrieved from PubMed. First, a small-scale microarray study performed with human native nasal epithelial cells (F508del homozygous patients vs. controls) was used [31]. We also retrieved genes from other papers aimed to compare gene expression in CF vs normal cells [32]. Another work using non-CF and F508del-CFTR homozygote samples, showed significant changes in the expression in 24 genes (two-sample t-test, p < 0.00001). A three-filter comparative analysis showed that 18 genes were significantly increased and 6 genes were decreased in CF relative to and non-CF samples, respectively [33]. We also retrieved genes from a microarray study in which results from 12 CF and 11 non-CF participants were used [34], and from data in which functional CFTR was absent of the plasma membrane [35]. Finally, we used a paper in which 4 studies evaluating the effect of the F508del-CFTR mutation on airway epithelial cells gene expression were analyzed [36]. A profiled gene expression in CF and non- CF nasal and bronchial epithelium samples, using Illumina HumanRef-8 Expression BeadChips was used. It showed that 863 genes were differentially expressed between CF and non- CF bronchial epithelium and that only 15 were differentially expressed between CF and non- CF nasal epithelium [37]. This indicated that within airways, gene expression varies depending on the region that is studied. Up- and down-regulated genes were compared within these studies [32–37] and common genes were searched (Table 1). We found that 75 genes were up-regulated and 114 were down-regulated in human airway cells expressing F508del-CFTR (Table 1, lower part).

Nonalcoholic fatty liver disease has a large spectrum ranging from simple steatosis to NASH, which may lead to progressive fibrosis. In the first study that we used [38], the abundance of intra-hepatic messenger RNA for a broad array of genes was measured. From this study we retrieved differentially expressed genes in NASH-vs-normal liver. Among 6,412 genes, only 16 were differentially expressed in subjects with NASH when compared with controls (Table 2). We used another study using microarrays [39]. 14 genes for NASH-vs-obese controls were found to be up-regulated (Table 2). In a third study, genes in NASH patients and controls were selected [40]. Whereas, 34 genes were differentially expressed in NASH vs non-obese controls, 19 of these genes had no significant differences in obese vs non-obese, suggesting a stronger association of these genes to NASH. Therefore, we used this list of 15 genes (Table 2). Finally, a study with normal, steatotis, NASH with fatty liver, and NASH without fatty liver samples was analyzed. Among 11,633 genes with altered expression out of 33,252 genes, 39 genes were changed in expression, between normal and NASH [21]. Thus, a list of 84 differentially expressed genes from 4 different studies was used in the present study (Table 2).

**Table 2:** Retrieved differentially expressed genes in Liver Disease (from [38–41]). When possible, names or abbreviations as well as OMIM accession number were added (in italic). The number sign # is used because α1-antitrypsin deficiency is caused by mutation in the SERPINA1 gene (OMIM: 107400). “?” is used when no result was obtained in our OMIM search for the corresponding gene name or when several results could be retrieved. The lower list is retrieved from [21] shows the Solute Carrier Family (SLC, sodium/potassium/chloride transporter family) with differential gene expression in NASH.

Gene name	NASH vs. controls	Reference
Aldehyde oxidase (AOX1, OMIM: 602841)	-2.4	
Cu/Zn SOD (SOD1, OMIM: 147450)	-3.7	
Catalase (CAT, OMIM: 115500)	-2.4	
Glucose-6 phosphatase (G6PC, OMIM: 613742)	-4.9	
Alcohol dehydrogenase (?)	-2.6	
Ubiquitin (?)	+2.6	
methylmalonate semialdehyde dehydrogenase (?)	-3.3	
Elongation factor TU (TUFM, OMIM: 602389)	-2.9	
HM CoA-synthase (COASY, OMIM: 609855)	-2.2	[38]
LCA CoA-synthetase (?)	-2.6	
MO CoA-thiolase (?)	-2.3	
a1-antitrypsin^#^	+3.7	
Complement C3 (C3, OMIM: 120700)	+3.3	
HFREP-1 (?)	+2.7	
Cytochrome P-450 4A (CYP4A11 OMIM: 601310)	-3.9	
MC-specific LRP (LRP1, OMIM: 107770)	-3.7	
POU2AF1 (POU Domain, Class 2, Associating Factor 1, OMIM: 601206)	+2.8	
MMP2 (Matrix Metalloproteinase 2, OMIM: 120360)	+2.4	
C-fos-induced growth factor/vascular endothelial growth factor D (FIGF, OMIM: 300091)	+2.2	
c12ORF14 (?)	+2.2	
CSPG2 (?)	+2.1	
Hypothetical protein dj462o23.2	+2.1	
ROD1 (Regulator of Differentiation 1, OMIM: 607527)	+2.1	[39]
STXBP1 (Syntaxin-Binding Protein 1, OMIM: 602926)	+2.1	
Serine dehydratase (SDS, OMIM: 182128)	+0.5	
SULT1A2 (Sulfotransferase Family 1A, OMIM: 601292)	+0.5	
CUTL2 (Cut-Like 2, OMIM: 610648)	+0.5	
ARL6IP (ADP-Ribosylation-Like Factor 6-Iinteracting Protein 1, OMIM: 607669)	+0.5	
Acyl-coenzyme A (CoA) dehydrogenase	+0.5	
Ornithine aminotransferase (OAT, OMIM: 613349)	+0.4	
NNMT (Nicotinamide N-methyltransferase, OMIM: 600008)	+0.3	
ODC1 (Ornithine Decarboxylase 1, OMIM: 165640)	+0.4	
IGFBP1 (Insulin-Like Growth Factor-Binding Protein 1, OMIM: 146730)	+0.1	
ATF3 (Activating Transcription Factor 3, OMIM: 603148)	+0.1	
CHI3L1 (Chitinas 3-Like 1, OMIM: 601525)	+0.4	
NFIL-3 (Nuclear Factor, Interleukin 3-Regulated, OMIM: 605327)	+0.4	
ZFP36 (Zinc Finger Protein 36, OMIM: 190700)	+0.3	
IL-15RA (Interleukin 15 Receptor, Alpha, OMIM: 601070)	+0.4	[40]
TGFb1 (Transforming Growth Factor, Beta-1, OMIM: 190180)	+0.4	
CAT (Catalase, OMIM: 115500)	+2.0	
GSTA4 (Glutathione S-Transferase, Alpha-4, OMIM: 605450)	+2.1	
LTF (Lactotransferrin, OMIM: 150210)	+2.0	
TF (transferrin, OMIM:190000)	+2.0	
ABR (Active BCR-Related Gene, OMIM: 600365)	+0.3	
PNRC1 (Proline-Rich Nuclear Receptor Coactivator 1, OMIM: 606714)	+0.3	
SULT1C4 (Sulfotransferase Family 1C, Member 4?)	1	
MTR (5-@Methyltetrahydrofolate-Homocysteine S Methyltransferase, OMIM: 156570)	1	
PAPSS2 (3’@Phosphoadenosine 5’Phosphosulfate Synthase2, OMIM: 603005)	-1	
CYP4F11 (Cytochrome P450, Family 4, Subfamily F, Polypeptide 11, OMIM: 611517)	-1	
ALDH4A1 (Aldehyde Dehydrogenase, Family 4, Subfamily A, Member 1, OMIM: 606811)	-1	
AOX1 (Aldehyde Oxidase 1, OMIM: 602841)	-1	
UGCG (UDP-Glucose Ceramide Glucosyltransferase, OMIM: 602874)	1	
CYP2E1 (Cytochrome P450, Subfamily IIE, OMIM: 124040)	-1	
GPX2 (Glutathione Peroxidase 2, OMIM: 138319)	-1	
HGSNAT (Heparan-Alpha-Glucosaminide N-Acetyltransferase, OMIM: 610453)	1	
FMO2 (Flavin-Containing Monooxygenase 2, OMIM: 603955)	1	
NNMT (Nicotinamide N-Methyltransferase, OMIM: 600008)	-1	
SULT1C2 (Sulfotransferase Family 1C, Member2, OMIM: 608357)	1	
HSD17B10 (17-@Beta-HydroxySteroid Dehydrogenase X, OMIM: 300256)	-1	
LOX (Lysyl Oxidase, OMIM: 153455)	1	
GGT5 (Gamma-GlutamylTransferase 5; OMIM: 137168)	1	
GSTP1 (Glutathione S-Transferase, PI, OMIM: 134660)	1	
PAPSS1 (3’@Phosphoadenosine 5’-Phosphosulfate Synthase 1, OMIM: 603262)	1	
MTRR (Methionine Synthase Reductase, OMIM: 602568)	-1	
CYP3A7 (Cytochrome P450, Subfamily IIIA, Polypeptide 7, OMIM: 605340)	1	
GCKR (Glucokinase Regulatory Protein, OMIM: 600842)	-1	[41]*
CYP7B1 (Cytochrome P450, Family 7, Subfamily B, Polypeptide 1, OMIM: 603711)	1	
PTGS2 (Prostaglandine-Endoperoxide Synthase 2, OMIM: 600262)	1	
CYP21A2 (Cytochrome P450, Family 21, Subfamily A, Polypeptide 2, OMIM: 613815)	-1	
HSD17B4 (17-@Beta-Hydroxysteroid Dehydrogenase IV, OMIM: 601860)	-1	
ALDH8A1 (Aldehyde Dehydrogenase 8 Family, Member A1, OMIM: 606467)	-1	
GCLM (Glutamate-Cysteine Ligase, Modifier Subunit, OMIM: 601176)	1	
NAT1 (N-Acetyltransferase 1, OMIM: 108345)	-1	
HNMT (Histamine N-Methyltransferase, OMIM: 605238)	1	
CYP51A1 (Cytochrome P450, Family 51, Subfamily A, Polypeptide 1, OMIM: 601637)	-1	
UCHL3 (Ubiquitin Carboxyl-Terminal Esterase L3, OMIM: 603090)	-1	
UGT2B4 (Uridine Diphosphate Glycosyltransferase 2 Family, Member B4, OMIM: 600067)	-1	
ALDH1A3 (Aldehyde Dehydrogenase 1 Family, Member A3, OMIM: 600463)	1	
CYB5R3 (Cytochrome b5 Reductase 3, OMIM: 613213)	-1	
HSD3B2 (3-@Beta-HydroxySteroid Dehydrogenase 2, OMIM: 613890)	-1	
BLVRA (Biliverdin Reductase A, OMIM: 109750)	1	
GZMA (Granzyme A, OMIM: 140050)	1	
UGT2B (?)	1	
CES1 (Carbosylesterase 1, OMIM: 114835)	-1	
Uptake transporter with Differential Gene Expression (from 41): SLC12A2 SLC39A14 SLC17A2 SLC44A1 SLC34A2 SLCO3A1 SLC25A36 SLCO2A1 SLC13A5 SLC22A15 SLC35B1 SLC4A2 SLC1A4 SLC31A1 SLC2A3 SLC2A10 SLC24A3 SLC33A1 SLC4A7 SLC5A1 SLC28A3 SLC25A24 SLC17A4 SLC44A3 SLC35F2 SLC38A10 SLC43A1 SLCO4C1 SLC22A3 SLC5A6 SLC35A2 SLC25A38 SLC6A11 SLC39A7 SLC1A1 SLC39A9 SLC2A14 SLC16A10 SLC22A23 SLC4A4 SLC41A2 SLC25A39 SLC38A11 SLC11A2 SLC16A4 SLC13A3 SLC6A6 SLC12A6 SLC24A6 SLC9A3R1 SLC23A1 SLC12A8 SLC46A1 SLCO1B3 SLC2A13 SLC5A4 SLC2A9 SLC38A6 SLC39A8 SLC43A3 SLC25A13 SLC35E1 SLC25A11 SLC25A12 SLC39A1 SLC39A6 SLC35C1 SLC35B4 SLC3A2 SLC30A5 SLC46A3 SLC25A34 SLC25A46 SLC10A7 SLC6A7 SLC9A8 SLC45A2 SLC25A17 SLC25A6 SLC45A1 SLC10A4 SLC22A2 SLC17A5 SLC26A6 SLC2A12 SLC39A3 SLC5A5 SLC20A1 SLC8A1 SLC26A1 SLC38A4 SLC25A33 SLC25A43 SLC6A9 SLC25A31 SLC37A3 SLC1A3 SLC27A2 SLC25A28 SLC24A2 SLC13A2 SLC1A5 SLC7A6 SLC39A10 SLC25A27 SLC5A12 SLC9A11 SLC29A4 SLC38A7 SLC25A37 SLC34A1 SLC6A15 SLC29A2 SLC17A7 SLC20A2 SLC18A3 SLC25A37 SLC9A9 LST-3TM12 SLC38A8 SLC22A8 SLC35B3 SLC16A13 SLC43A2 SLC25A20 SLC22A6 SLC22A13 SLC14A2 SLC25A41 SLC35F1 SLC37A1 SLC4A5 SLC27A6 SLC32A1 SLC44A2 SLC5A10 SLC26A5 SLC7A9 SLC9A1 SLC22A11 SLC25A23 SLC25A44 SLC30A1 SLC7A3 SLC17A8 SLC6A5 SLC25A42 SLC25A2 SLC39A4 SLC25A35 SLC36A3 SLC45A4 SLC9A3R2 SLC6A3 SLC25A18 SLC9A7 SLC2A6 SLC45A3 SLCO6A1 SLC25A22		

*: 0 = No change, +1 = Up regulation, -1 = Down regulation.

For pancreatic disease, a study with normal and Chronic Pancreatitis specimens was used [43]. Comparison of the expression of 5,600 genes between the normal and Chronic Pancreatitis was performed. GenBank accession numbers of 152 genes with increased expression ([43], Footnote 1) and 34 with decreased genes levels in Chronic Pancreatitis ([43], Footnote 2) were retrieved. We analyzed those genes one by one and removed non human genes and pseudo genes. This led us to list 129 increased genes and 23 decreased genes in *Chronic Pancreatitis* (Table 3). Because 152 genes were simultaneously increased in pancreatic cancer, only 5 of 5,600 genes were significantly over expressed in *Chronic Pancreatitis* compared with normal pancreas: Mucin-6 (GenBank Accession Number: L07517), COMP (GenBank Accession Number: L32137), TPSB1 (GenBank Accession Number: M33493), Rearranged Ig-lambda light chain (GenBank Accession Number: X57809) and CRISP-3 (GenBank Accession Number: X95240). An analysis of 6,800 different genes expressed in samples of normal pancreas and *Chronic Pancreatitis* was studied [44]. 107 genes were predicted to be expressed within cells with *Chronic Pancreatitis* ([44], Table 1). Genes from both studies [43 and 44] were retrieved and non human, pseudo genes and common genes were withdrawn. Finally, we found 23 decreased genes and 229 increased genes in *Chronic Pancreatitis*, when compared to normal pancreas (Table 4).

**Table 3:** Retrieved differentially expressed genes in Chronic Pancreatitis (from [43]). Some genes were withdrawn according to the reasons explained in Methods.

LGALS4, ARPC1B, PMP22, POSTN, CDH11, IQGAP1, HLA-A, LGMN, AP2M1, SERPINH1, FCGBP, DYRK1A, PXDN, COL5A1, SPARC, LYZ, LGALS1, TCN1, CXCR4, TFF3, THBS2, RGS2, IGFBP5, COMP, PECAM1, FHL2, THY1, TPM2, JCHAIN, CD74, F13A1, CEACAM6, CYBA, PLA2G2A, CTSA, COL4A2, HBB, F3, CEACAM5, MGMT, XRCC5, HLA-DRB1, PSMC3, GBP2, HLA-DPB1, TNFRSF1A, IGFBP4, NR2F2, CFHR1, TGFBI, TAGLN, HSPG2, CALD1, COL1A1, PPIC, SEPT7, LCN2, FSCN1, CETN1, MDK, CAPG, COL1A2, IGHG3, ALDH1A3, ATP2A3, TPBG, GUCY1B3, ACVR1, IFITM2, COL3A1, FN1, TFF1, CDH5, HLA-F, GSN, ASS1, GLRX, HIF1A, LUM, JUNB, AOC3, VCAN, LAMB3, GPNMB, POLR2A, EMP1, TRIM22, PRMT1, ITGA1, MALL, ATP6V0D1, SNRPD3, NNMT, PYGB, ICAM3, GPX2, COL6A3, AGPS, ITGB2, SGSH, LOXL1, RARRES1, C3AR1, HLA-DRA, TAX1BP3, AKAP12, LITAF, CCL19, ABCC8, FILIP1L, GRN, KRT19, ABCG1, SRGN, FLNA, CRISP3, THBS1, VASP, PTPRC, S100A13, ACTA2, CD14, IGHM, COL4A2, PKM, MMP1, NFE2L1, HLA-E, LCN2	**129 Genes expressed at increased level**
FGL1, FUT1, PCDHB11, PCK1, TKT, AOX1, TEP1, BNIP3, ART1, VHL, SPINK1, EPHX1, SELENBP1, SLC25A1, SLC14A1, TYMP, PEX7, BTN3A1, FASN, GSTA2, ANPEP, ELANE, HOXB2	**23 Genes expressed at decreased level**

**Table 4:** Combined differentially expressed genes in Chronic Pancreatitis [43, 44]. Some genes were withdrawn according to the reasons explained in Methods. OMIM ID and full names were searched for each gene. 23 genes were found to be decreased and 229 genes were found to be increased in Chronic Pancreatitis (p<0.05).

**Genes expressed at increased levels in Chronic Pancreatitis**	**OMIM ID**	**Reference**
LGALS4 (Lectin, Galactoside-Binding, Soluble, 4)	602518	
ARPC1B (actin related protein 2/3 complex subunit 1B)	604223	
PMP22 (peripheral myelin protein 22)	601097/608777	
POSTN (periostin, osteoblast specific factor)	600023	
CDH11 (cadherin 11)	603379	
IQGAP1 (IQ motif containing GTPase activating protein 1)	142800	
HLA-A (major histocompatibility complex, class I, A)	602620	
LGMN (legumain)	601024	
AP2M1 (adaptor related protein complex 2 mu 1 subunit)	600943	
SERPINH1 (serpin peptidase inhibitor, clade, member 1)	601437	
FCGBP (Fc fragment of IgG binding protein)	600855	
DYRK1A (dual specificity tyrosine phosphorylation regulated kinase 1A)	605158	
PXDN (peroxidasin)	120215	
COL5A1 (collagen type V alpha 1)	182120	
SPARC (secreted protein acidic and cysteine rich)	153450	
LYZ (lysozyme)	150570	
LGALS1 (lectin, galactoside-binding, soluble 1)	189905	
TCN1 (transcobalamin 1)	162643	
CXCR4 (C-X-C motif chemokine receptor 4)	600633	
TFF3 (trefoil factor 3)	188061	
THBS2 (thrombospondin 2)	600861	
RGS2 (regulator of G-protein signaling 2)	146734	[43]
IGFBP5 (insulin like growth factor binding protein 5)	600310	
COMP (cartilage oligomeric matrix protein)	173445	
PECAM1 (platelet/endothelial cell adhesion molecule 1)	602633	
FHL2 (four and a half LIM domains 2)	188230	
THY1 (Thy-1 cell surface antigen)	190990	
TPM2 (tropomyosin 2 beta)	147790	
JCHAIN (joining chain of multimeric IgA and IgM)	142790	
CD74 (CD74 molecule)	134570	
F13A1 (coagulation factor XIII A chain)	163980	
CEACAM6 (carcinoembryonic antigen related cell adhesion molecule 6)	608508	
CYBA (cytochrome b-245 alpha chain)		
PLA2G2A (phospholipase A2 group IIA)	172411	
CTSA (cathepsin A)	613111	
COL4A2 (collagen type IV alpha 2)	120090	
HBB (hemoglobin subunit beta)	141900	
F3 (coagulation factor III, tissue factor)	134390	
CEACAM5 (carcinoembryonic antigen related cell adhesion molecule 5)	114890	
MGMT (O-6-methylguanine-DNA methyltransferase)	156569	
XRCC5 (X-ray repair compl. defective repair in Chinese hamster cells 5)	194364	
HLA-DRB1 (major histocompatibility complex, class II, DR beta 1)	142857	
PSMC3 (proteasome 26S subunit, ATPase 3)	186852	
GBP2 (guanylate binding protein 2)	600412	
HLA-DPB1 (major histocompatibility complex, class II, DP beta 1)	142858	
TNFRSF1A (tumor necrosis factor receptor superfamily member 1A)	191190	
IGFBP4 (insulin like growth factor binding protein 4)	146733	
NR2F2 (nuclear receptor subfamily 2 group F member 2)	107773	
CFHR1 (complement factor H related 1)	134371	
TGFBI (transforming growth factor beta induced)	601692	
TAGLN (transgelin)	600818	
MDK (midkine, neurite growth-promoting factor 2)	162096	
CAPG (capping actin protein, gelsolin like)	153615	
COL1A2 (collagen type I alpha 2)	120160	
IGHG3 (immunoglobulin heavy constant gamma 3)	147120	
HSPG2 (heparan sulfate proteoglycan 2)	142461	
CALD1 (caldesmon 1)	114213	
COL1A1 (collagen type I alpha 1)	120150	
PPIC (peptidylprolyl isomerase C)	123842	
SEPT7 (septin 7)	603151	
LCN2 (lipocalin 2)	600181	
FSCN1 (fascin actin-bundling protein 1)	602689	
CETN1 (centrin 1)	603187	
ALDH1A3 (aldehyde dehydrogenase 1 family member A3)	600463	
ATP2A3 (ATPase sarcoplasmic/endoplasmic reticulum Ca2+ transporting 3)	601929	
TPBG (trophoblast glycoprotein)	190920	
GUCY1B3 (guanylate cyclase 1, soluble, beta 3)	139397	
ACVR1 (activin A receptor type 1)	102576	
GLRX (glutaredoxin)	600443	[43]
HIF1A (hypoxia inducible factor 1 alpha subunit)	603348	
LUM (lumican)	600616	
JUNB (jun B proto-oncogene)	165161	
AOC3 (amine oxidase, copper containing 3)	603735	
VCAN (versican)	118661	
LAMB3 (laminin subunit beta 3)	150310	
GPNMB (glycoprotein nmb)	604368	
POLR2A (polymerase (RNA) II subunit A)	180660	
EMP1 (epithelial membrane protein 1)	602333	
TRIM22 (tripartite motif containing 22)	606559	
PRMT1 (protein arginine methyltransferase 1)	602950	
ITGA1 (integrin subunit alpha 1)	192968	
MALL (mal, T-cell differentiation protein-like)	602022	
ATP6V0D1 (ATPase H+ transporting V0 subunit d1)	607028	
SNRPD3 (small nuclear ribonucleoprotein D3 polypeptide)	601062	
NNMT (nicotinamide N-methyltransferase)	600008	
PYGB (phosphorylase, glycogen; brain)	138550	
ICAM3 (intercellular adhesion molecule 3)	146631	
GPX2 (glutathione peroxidase 2)	138319	
COL6A3 (collagen type VI alpha 3)	120250	
AGPS (alkylglycerone phosphate synthase)	603051	
ITGB2 (integrin subunit beta 2)	600065	
SGSH (N-sulfoglucosamine sulfohydrolase)	605270	
LOXL1 (lysyl oxidase like 1)	153456	
RARRES1 (retinoic acid receptor responder 1)	605090	
C3AR1 (complement component 3a receptor 1)	605246	
HLA-DRA (major histocompatibility complex, class II, DR alpha)	142860	
TAX1BP3 (Tax1 binding protein 3)	616484	
AKAP12 (A-kinase anchoring protein 12)	604698	
LITAF (lipopolysaccharide induced TNF factor)	603795	
CCL19 (C-C motif chemokine ligand 19)	602227	
ABCC8 (ATP binding cassette subfamily C member 8)	600509	
FILIP1L (filamin A interacting protein 1-like)	612993	
GRN (granulin)	138945	
KRT19 (keratin 19)	148020	
ABCG1 (ATP binding cassette subfamily G member 1)	603076	
SRGN (serglycin)	177040	
FLNA (filamin A)	300017	
CRISP3 (cysteine rich secretory protein 3)	600824	
THBS1 (thrombospondin 1)	188060	
VASP (vasodilator-stimulated phosphoprotein)	601703	
PTPRC (protein tyrosine phosphatase, receptor type C)	151460	
S100A13 (S100 calcium binding protein A13)	601989	
ACTA2 (actin, alpha 2, smooth muscle, aorta)	102620	
CD14 (CD14 molecule)	158120	
IGHM (immunoglobulin heavy constant mu)	147020	[43]
COL4A2 (collagen type IV alpha 2)	120090	
PKM (pyruvate kinase, muscle)	179050	
MMP1 (matrix metallopeptidase 1)	120353	
IFITM2 (interferon induced transmembrane protein 2)	605578	
COL3A1 (collagen type III alpha 1)	120180	
FN1 (fibronectin 1)	135600	
TFF1 (trefoil factor 1)	113710	
CDH5 (cadherin 5)	601120	
HLA-F (major histocompatibility complex, class I, F)	143110	
GSN (gelsolin)	137350	
ASS1 (argininosuccinate synthase 1)	603470	
NFE2L1 (nuclear factor, erythroid 2 like 1)	163260	
HLA-E (major histocompatibility complex, class I, E)	143010	
LCN2 (lipocalin 2)	600181	
ACTA2 (actin, alpha 2, smooth muscle)	102620	
AEBP1 (AE-binding protein)	602981	
ALDH2 (aldehyde dehydrogenase 2, mitochondrial)	100650	
AOC3 (amine oxidase, copper containing 3)	603735	
APOC1 (apolipoprotein C-I)	107710	
APOE (apolipoprotein E)	107741	
BTN3A1 (butyrophilin, subfamily 3, member A1)	613593	
BTN3A2 (butyrophilin, subfamily 3, member A2)	613594	
CDH11 (cadherin 11)	600023	
CALD1(caldesmon 1)	114213	
CTSC (cathepsin C)	602365	
CTSK (cathepsin K)	601105	
CTSS (cathepsin S)	116845	[44]
CD37 (CD37 molecule)	151523	
CD74 (CD74 molecule)	142790	
FOXN3 (forkhead box N3)	602628	
CCR1(chemokine (C-C motif) receptor 1)	601159	
CXCR4 (chemokine (C-X-C motif), receptor)	162643	
CHN1 (chimerin 1)	118423	
COL1A1(collagen type I, alpha 1)	120150	
COL1A2 (collagen type I, alpha 2)	120160	
COL3A1 (collagen type III, alpha 1)	120180	
COL4A2 (collagen type IV, alpha 2)	120090	
COL5A2 (collagen type V, alpha 2)	120190	
COL6A3 (collagen type VI, alpha 3)	120250	
COL10A1(collagen type X, alpha 1)	120110	
C1R (complement component 1, r subcomponent)	613785	
C1S (complement component 1, s subcomponent)	120580	
C2 (complement component 2)	613927	
CBFB (core-binding factor, beta subunit)	121360	
CDKN1C (cyclin-dependent kinase inhibitor 1C)	600856	
DPYSL2 (dihydropyrimidinase-like 2)	602463	
DPYSL3 (dihydropyrimidinase like 3)	601168	
ENTPD1(ectonucleoside triphosphate diphosphohydrolase 1)	601752	
ENPP2 (ectonucleotide pyrophosphatase/phosphodiesterase)	601060	
EDNRA (endothelin receptor type A)	131243	
FCER1G (Fc fragment of IgE, high-affinity I, receptor)	147139	
FBN1 (fibrillin 1)	134797	
FAP (fibroblast activation protein, alpha)	600403	
FN1(fibronectin 1)	135600	
FOLR1 (folate receptor)	136430	
GSN (gelsolin)	137350	
GPNMB (glycoprotein (transmembrane) nmb)	604 368 600 784	
GZMK (granzyme K)	142995	
HLX (H2.0-like homeobox)	602179	
HSPB2 (heat-shock 27-KD protein 2)	603348	
HIF1A (hypoxia-inducible factor 1, alpha subunit)	188061	
THBS2 (thrombospondin 2)	188230	
THY1 (thy-1 cell surface antigen)	602272	
TCF4 (transcription factor 4)	600818	
TAGLN (transgelin)	600769	
TSPAN8 (tetraspanin 8)	602529	
TUBA1A (tubulin alpha 1a)	601693	
UCP2 (uncoupling protein 2 (mitochondrial, proton carrier))	604078	
ZNF136 (zinc finger protein 136)	147120	
IGHG3 (immunoglobulin heavy constant gamma 3 (G3m marker))	147200	
IGKC (immunoglobulin kappa constant)	192968	
ITGA1 (integrin subunit alpha 1)	135620	[44]
ITGA5 (integrin subunit alpha 5)	120980	
ITGAM (integrin subunit alpha M)	146933	
IL10RA (Iinterleukin-10 receptor, alpha)	150390	
LTBP1 (latent transforming growth factor beta binding protein 1)	600616	
LUM (lumican)	109170	
LST1 (leukocyte specific transcript 1)	600978	
LTB (lymphotoxin beta)	601476	
LAPTM5 (lysosomal protein transmembrane 5)	153455	
LOX (lysyl oxidase)	153456	
LOXL1 (lysyl oxidase-like 1)	143010	
HLA-E (major histocompatibility complex, class I, E)	142855	
HLA-DMA (major histocompatibility complex, class II, DM alpha)	142856	
HLA-DMB (major histocompatibility complex, class II, DM beta)	142857	
HLA-DRB1 (major histocompatibility complex, class II, DR beta)	120360	
MMP2 (matrix metalloproteinase 2)	602281	
MFGE8 (milk fat globule-EGF factor 8 protein)	600922	
MYLK (myosin light chain kinase)	608243	
NDN (necdin, MAGE family member)	600613	
NBL1 (neuroblastoma, suppression of tumorigenicity)	601488	
NCF4 (neutrophil cytosolic factor 4)	600008	
NNMT (nicotinamide N-methyltransferase)	605399	
NID2 (nidogen 2)	608777	
POSTN (periostin, osteoblast specific factor)	607332	
NREP (neuronal regeneration related protein)	601097	
PMP22 (peripheral myelin protein 22)	153430	
LCP1 (lymphocyte cytosolic protein 1)	173445	
PECAM1 (platelet/endothelial cell adhesion molecule)	173490	
PDGFRA (platelet derived growth factor receptor alpha)	173410	
PDGFRB (platelet-derived growth factor receptor, beta polypeptide)	600270	
PCOLCE (procollagen C-endopeptidase enhancer)	602194	
HTRA1 (HtrA serine peptidase 1)	176970	
PRKCB (protein kinase C beta)	151460	
PTPRC (protein tyrosine phosphatase, receptor type, C)	601890	
PTK7 (protein tyrosine kinase 7 (inactive))	609492	
RASSF2 (Ras association domain family member 2)	602189	[44]
RGS3 (regulator of G-protein signalling 3)	182120	
SPARC (secreted protein, acidic, cysteine rich)	107400	
SERPINA1 (serpin peptidase inhibitor, clade A1)	106150	
AGT (angiotensinogen)	173360	
SERPINE1 (Serine (or cysteine) proteinase inhibitor, clade E, member 1)	313020	
SAT1 (spermidine/spermine N1-acetyltransferase)	601737	
SMARCD3 (SWI/SNF related, matrix associated, actin dependent regulator of chromatin, subfamily d, member 3)		
**Genes expressed at decreased levels in Chronic Pancreatitis**	**OMIM ID**	
FGL1 (fibrinogen like 1)	605776	
FUT1 (fucosyltransferase 1 (H blood group))	211100	
PCDHB11 (protocadherin beta 11)	606337	
PCK1 (phosphoenolpyruvate carboxykinase 1)	614168	
TKT (transketolase)	606781	
AOX1 (aldehyde oxidase 1)	602841	
TEP1 (telomerase associated protein 1)	601686	
BNIP3 (BCL2/adenovirus E1B 19kDa interacting protein 3)	603293	
ART1 (ADP-ribosyltransferase 1)	601625	
VHL (von Hippel-Lindau tumor suppressor)	608537	[43]
SPINK1 (serine peptidase inhibitor, Kazal type 1)	167760	
EPHX1 (epoxide hydrolase 1)	132810	
SELENBP1 (selenium binding protein 1)	604188	
SLC25A1 (solute carrier family 25 member 1)	190315	
SLC14A1 (solute carrier family 14 member 1)	613868	
TYMP (thymidine phosphorylase)	131222	
PEX7 (peroxisomal biogenesis factor 7)	601757	
BTN3A1 (butyrophilin subfamily 3 member A1)	613593	
FASN (fatty acid synthase)	600212	
GSTA2 (glutathione S-transferase alpha 2)	138360	
ANPEP (alanyl aminopeptidase, membrane)	151530	
ELANE (elastase, neutrophil expressed)	130130	
HOXB2 (homeobox B2)	142967	

### Differential gene expressions retrieved from microarray studies

We also acquired gene array data from the GEO database, a public available archive of individual microarrays studies [46, 47]. GSE40445 [31], is a study of gene expression in human native nasal epithelial cells from F508del-CFTR homozygous patients and non-CF controls. GSE48452 [48] is an expression profiling performed with samples of control human liver, healthy obese, steatosis and NASH). Only controls and NASH groups were re-analyzed here. For PI, we failed to find any array analysis. Therefore, we used a gene expression of type 1 diabetes-vs-classical type 1A diabetes-vs-healthy controls array (GSE44314) [49]. We solely re-analyzed classical type 1A diabetes-vs-healthy controls. 246, 211 and 145 genes were retrieved from our GSE40445, GSE48452 and GSE44314 re-analysis, respectively (Table 5). A Venn diagram was drawn (not shown) but no common genes among the genes selected from the 3 studies were found. Therefore, if there are some common genes in the 3 studies, they do not appear among the most highly regulated genes of each study.

**Table 5:** List of the genes retrieved from SE40445, GSE48452 and GSE44314. Genes that were found in at least two groups are underlined.

BANF1, EPHX1, CAB39, C2orf17, PLD3, NUCB2, SOC, PDXP, FRMD4B, ETS2, C20orf98, SLC33A1, TP53AP1, TJP1, TCEA2, AES, TETRAN, LOC348262, IFRD1, DSCR1L2, C10orf32, MGC5306, MGC20806, C16orf33, LNPEP, PHACTR2, CLDN1, RHBDL7, INSIG1, KIAA1847, ATP6V0A4, PTDSS2, DKFZP761D0211, TAF11, MRPL40, TNRC5, MAOB, KIAA1434, COBRA1, APBA2BP, GNA11, DKFZp434I1610, ARL8, SDK1, GSDMDC1, IFNGR2, TCTA, EP300, ARFGAP3, TOMM34, TTC13, HINT2, RPS6KB1, FEM1A, ERAL1, NUDT14, CCND3, RAB22A, MYCPBP, SEC14L3,CDR2, KIAA1033, RBM21, ARID1A, TTC14, ZADH1, DNCLI2, VARS2, HSPC268, TMC3, ENPP5, PBP, DREV1, C14orf31, TCERG1, CPNE2, LOC203069, B3GAT3, GSTM1, DUSP10, MB, RNF152, HSCARG, AP1G1, FLJ32096, C12orf8, LOC124446, DCTN1, APG16L, DGCR6, LOC144438, LOC90378, CUEDC2, ARRDC3, ZNF562, WDR13, EVA1, C7orf24, MECT1, FLNC, G6PC3, GRCC9, CHFR, KLF3, DKFZP564O243, ZBTB34, POLK, ENTPD4, TMC4, IFI16, PYCR2, PEX14, C6orf18, SIAT7E, UBE2M, DDX26, DKFZP586B1621, GSTM2, SP192, MGC4172, SIGIRR, CD44, FBXO28, CD19, GNB2, LOC51234, LY6D, MRPL4, NRD1, C10orf137, TSC1, PRDX5, PYCRL, PLA2G10, RABL2A, CGI-14, PIP5K3, MEA, ZNF297B, LRP16, DISP2, MLPH, TRAPPC1, HSPBP1, COG5, CREBBP, PHGDH, MGC15416, CCNE2, C6orf93, CAPS, MYO9A, KHSRP, IL12A, BDP1, FLJ13111, THAP7, LOC388389, FLJ13798, FLJ14566, TMEM1, ACO2, CUL5, LOC388962, TJP3, TPRA40, KIAA1145, NPC1, LOC389072, FLJ20406, PRNPIP, C11orf2, MGC45438, E4F1, DPM3, PTPRN2, PARD3, C12orf10, C6orf102, FLJ23342, CTSD, AP2B1, MOSPD3, CECR1, APCDD1, C6orf108, EDEM1, RUNX2, FDXR, ITGAV, SPPL2B, DHX15, GALNT7, GDI1, MID1IP1, SLC25A10, LGP1, YT521, MAP1LC3A, ALDH3B1, RALGDS, LOC285458, TFCP2L4, MLL5, IGFBP2, SMAP1, TRIM32, C20orf149, C20orf28, TGFBR2, MRPL49, RAPGEF6, DUSP8, SH3GLB1	**Genes obtained from SE40445**
LYZ, CDH23, SFMBT2, LOC654342/LOC645166, PECAM1, HLA-DRA, ZFP1, TLR7, KRR1/GLIPR1, ARHGAP31, GPRC5C, DPYSL2, GRTP1, GMFG, IFI16, PIP4K2A, ACSS1, MYO5A, STXBP1, PLA2G4A, H2AFY2, SYK, UROC1, SEMA3D, AMICA1, MSN, LAMA4, ZEB2, DHTKD1, TMEM144, INS-IGF2, EPHA3, ENG, OGFRL1, SYP, F2R, ABR, GPNMB, GCDH, PIEZO2, SLC38A1, CSF2RA, TNFAIP8, ABAT, CCND2, GCAT, NIN, FRZB, COL14A1, CA14, GUCY1A3, MRAS, NCF2, SULF2, ITGA9, HLA-DMA, PLXDC2, MYOF, TPM4, LOC100130691, FOXI2, SAMD9L, SCPEP1, SEL1L3, IQGAP1, APOBEC3C, TMSB10, ALOX5, ALKBH7, ENTPD1, PODXL, CD74, IGK/IGKV3-20/IGHV3-23, LCP1, SLC6A6, SUN2, FABP4, TANC1, PPM1K, PTGS1, ZNF532, CLEC10A, CPLX1, CNTNAP3B/CNTNAP3, AADAT, H2AFY, FCGR2C/FCGR2B, DOCK11, MAPRE1, DOCK10, CACHD1, YBX3, IGKC, FABP5, SPARC, RASGRP1, GLS2, GUCY1B3, KLF15, FLRT2, FCGR2A, TMEM120A, LSP1, HIST2H2BF/HIST2H2BA, SLC13A4, PIK3R3, AHCY, EBPL, ADAMTSL2, ACVRL1, MECOM, ELMO2, FANCI, HEG1, RNASE1, ARL3, ACKR2, SARDH, SLIT2, PTPRCAP, MTHFD1L, SGCB, ACER3, PDGFRB, PXMP2, SPP1, ASAP1, CMTM7, FMNL3, CD37, LGALS3, IGHG1/IGHV3-48, FAM20C, COL3A1, NPNT, HLA-DPB1, SSBP2, MTHFD2, IGHG1/IGHD, UTRN, CENPV, HDAC7, HSD17B11, RAB31, SCFV/IGHV4-31/GHM/IGHG1/IGHV3-30, MAML2, PCK2, SNX29, RASAL2, RHOJ, CD48, NAV1, MFSD6, ARHGEF6, PSPN, MFAP4, GYS2, WDR87, HLA-DRB4/HLA-DRB1, CD44, HIST1H1B, UG0898H09, GPR124, DZIP1/CLDN10, DPT, PRKX, SLC39A10, COL12A1, SCRN1, ARMCX6, GPR174, ITGAX, LAIR1, JAM2, IMP3, DOCK8, TSPAN3, GSN, PKM, IGHG4/IGHA2, IGF1, GLT8D2, HLA-DPA1, FOCAD, SUSD1, HIST1H3B, PIGV, STK39, MGMT, PREX1, PTPRM, TCF4, KRTAP4-7/KRTAP4-9/KRTAP4-8/KRTAP4-6, FAM65B, AMDHD1, FAM8A1, CRYBG3/MINA, DOCK2, NOD1, PMP22, LOC100134868, TMEM165, ANKRD36/ANKRD36B, SORT1, FAR1, INPP5D, ACOT12, PTPLAD2, VIM, PTPN13, IKBKE	**Genes obtained from GSE48452**

### Common genes in CF, retrieved from bibliography and microarray studies

We then search for common genes between the genes which were obtained by the bibliographic search for CF genes (Table 1), Liver Disease (Table 2) and *Chronic Pancreatitis* (Tables 3 and 4) and the genes which were retrieved by the re-analyzing of GSE40445, GSE48452 and GSE44314. The result is summarized in Table 6.

**Table 6:** Genes involved in CF, Chronic Pancreatitis and Liver Disease. Common genes present in our re-analyzed data of the GEO database and in our bibliographic analysis. Common genes in CF and in pancreatic and in liver affections are underlined.

		Bibliographic analysis		
		Differentially expressed genes in CF	Differentially expressed genes in Chronic Pancreatitis	Differentially expressed genes in Liver Disease
**GEO Analysis**	**CF-vs-non CF re-analysis of SE40445**	IFRD1* [32–35, 37] **IFI16** [32–35, 37] **CCNE2** [32] **IGFBP2** [33, 35]	**EPHX1** [38]	**SLC33A1** [41]
	**Diabetes-vs-healthy re-analysis of GSE44314**	HLA-DRB1** [32–35, 37] **DSP** [32–35, 37] **HLA-DQA1** [34]	HLA-DRB1** [43, 44] CTSA [43] PKM [43]	SLC25A36 [41] TGFB1*** [40]
	**NASH-vs-healthy re-analysis of GSE48452**	**HLA-DRA** [32–35, 37] **GPNMB** [32–35, 37] **NCF2** [32–35, 37] **RASGRP1** [32–35, 37] **LGALS3** [32–35, 37] **PTPN13** [32–35, 37]	LYZ [43] PECAM1 [43, 44] HLA-DRA [43] DPYSL2 [44] GPNMB [43, 44] HLA-DMA [44] IQGAP1 [43] ENTPD1 [44] CD74 [43, 44] LCP1 [44] IGKC [44] SPARC [43, 44] GUCY1B3 [43] PDGFRB [44] CD37 [44] COL3A1 [43, 44] HLA-DPB1 [43] GSN**** [43, 44] MGMT [43] TCF4 [44] PMP22 [43, 44]	STXBP1 [39] ABR [40] SLC6A6 [41] SLC39A10 [41]

Underlined: Genes that were found in the bibliographic analysis for CF and Chronic Pancreatitis genes.

*: also found in our OMIM search and common within CF, CF associated genes and Liver disease keywords

**: also found in our CTD search and common within Pancreatic Diseases and Liver Diseases keywords

***: also found in our OMIM search and is common within CF, CF associated genes, Liver disease and PI keywords and in CTD search for CF, Pancreatic Diseases and Liver Disease keywords

****: also found in common our OMIM search and common within CF, CF associated genes keywords

Only 4 genes were found to be common to both groups of genes retrieved from the bibliographic analysis and the GEO database analysis, regarding CF. These genes were IFRD1 (Interferon-related developmental regulator 1, OMIM Accession ID: 603502), IFI16 (Interferon gamma inducible protein 16, OMIM Accession ID: 147586), CCNE2 (Cyclin E2, OMIM Accession ID: 603775) and IGFBP2 (Insulin-like growth factor-binding protein 2, OMIM Accession ID: 146731). This low number of common genes is likely due to the fact that in microarray experiments, the recorded intensities depend on the conditions under which the measurements are made [50].

IFRD1, a histone deacetylase (HDAC)-dependent transcriptional co-regulator expressed during terminal neutrophil differentiation, was already identified of as a modifier gene in CF [51]. It was also found to be common for our CF, CF associated genes and Liver disease keywords search in OMIM.

We failed to find published works from PubMed, showing that IFI16, CCNE2 and IGFBP2 are also possibly involved in CF. Therefore, we searched in previously published works some lines of evidence to link these genes to CF.

IFI16 has a possible role in chronic inflammatory autoimmune disorders. The IFI16 protein is normally expressed in nuclei but may be mislocalized in the cytoplasm and secreted. Indeed, significant levels of extracellular IFI16 protein have been identified in the sera of patients with autoimmune diseases [52]. These data provide evidence for a function of IFI16 upon inflammation and tissue damage, as it is also observed in CF.

The human CCNE2 gene encodes a 404-amino-acid protein, related to cyclin E. It is associated with Cdk2 in a functional kinase complex [53] and is involved in the G1 progression [54]. Activated neutrophils elastase is found in high concentrations in the airways of CF patients [55] and has injurious effects on airway epithelial cells. Because the treatment of normal human bronchial epithelial by elastase results in G arrest, due to cyclin E complex inhibition ([56]), we propose that CCNE2 is possibly involved in the pathophysiology of CF.

The insulin-like growth factors (IGF) stimulates growth of multiple cell types. IGF-II access to the cell surface receptor is mediated by IGFBP-2 [57]. It was previously described that serum IGF-II levels were significantly lower in CF patients than in controls, whereas serum IGFBP-2 was significantly higher [58]. IGF-II/IGFBP-2 molar ratios were also found to be significantly lower in CF. Because chronic inflammation is an important modulator of the IGF/IGFBP system in CF and because IGF2 promotes development of pancreatic beta cells, related to some forms of diabetes mellitus, we also propose that IGFBP-2 is a modifier gene in CF.

Candidate genes in CF were selected due to their involvement in CF-related diseases or in similar diseases. Published studies have mostly examined genes involved in immune or inflammatory response (for review: [59]). These genes are: ACE, ADRβ2, ATB, CAPN10, ClCN2, DEFβ4, ENaC, FcβRII, GCLC, GSTM1, GSTM3, GSTP1, GSTT1, HFE, HLA-I, HLA-II, HLA-III, HSP70, IFN-γ, IL1-β, IL6, IL10, IL18, KCNJ11, MBL2, MIF, NOS1, NOS3, MASP-2, PPARβ, SERPINA1, SERPINA3, SFTPA1, SFTPA2, TLR4, TGFB1, TNFα, TNFα-receptor, and TNFβ [59]. Among the published modifier genes [59], we found using databases that TGFB1, SERPINA1, TNFα-receptor, GSTP1, ADRβ2, GSTM1, GSTT1 and TNFα are also involved in pancreatic and liver diseases (Figs 3 and 4). Among candidate gene modifiers of clinical phenotypes in CF, we also retrieved MBL2, EDNRA, TGFB1, IFRD1, IL8, MSRA, ADIPOR2, TCF7L2 and SERPINA1 (for review: [60]). When compared to our database search, we found that TGFB1 was present in OMIM and CTD and that IFRD1 and SERPINA1 were only present in OMIM, indicating that they are also possibly involved in pancreatic and liver diseases. IFRD1 was also found in our CF-vs-non CF re-analysis of SE40445 (Table 6).

In conclusion, we propose here that IFI16, CCNE2 and IGFBP2 are new candidates of modifiers genes in CF.

#### Common genes in CF and *Chronic Pancreatitis* and in CF and Liver Disease retrieved from bibliography and microarray studies

Common genes in CF-vs-non CF re-analysis of SE40445 and bibliographic search for *Chronic Pancreatitis* and Liver Disease were EPHX1 (Epoxide Hydrolase 1 Microsomal, OMIM Accession ID: 132810) and SLC33A1 (Solute carrier family 33 (Acetyl-CoA Transporter), member 1, OMIM Accession ID: 603690), respectively.

EPHX1 gene encodes a ubiquitous enzyme (α/β hydrolases), localized in the ER. Mutations in the EPHX1 gene contribute to several diseases [61] in link with tobacco exposure and lung carcinoma [62]. Regarding CF, a low activity of EPHX1 was found to be a risk factor for chronic obstructive pulmonary disease (COPD) in Caucasian [63]. Genetic polymorphisms of GSTP1 and EPHX1 were shown to correlate with oxidative stress markers and lung function in COPD [64]. It has to be noticed that GSTP1 belongs to our list of common genes for PI, CF and Liver Disease search In HuGE Navigator. Mutations in EPHX1 were also suggested to modify the severity of respiratory disorders in CF [65].

SLC33A1 (AT1) is a transporter of the ER membrane permitting the entry of acetyl-CoA into the ER lumen. Its down-regulation induces autophagy and cell death [66]. CFTR needs N-linked glycosylation. Its altered glycosyalation leads to a misfolded protein such as F508del-CFTR which is degradated [67]. The accumulation of this incorrectly folded protein in the ER, triggers the Unfolded Protein Response (UPR; [68, 69]). In CF, excessive UPR may be alleviated by the inhibition of ATF6 which possesses an endogenous inhibitor: XBP1 [69, 45] which controls ERAD by activating the expression of SLC33A1 [70]. Therefore, we propose here that SLC33A1 is likely a strong modulator in CF.

Differentially expressed genes in the bibliographic search for CF and in the Diabetes-vs-healthy re-analysis of GSE44314 were the Major Histocompatibility Complex Class II genes DRB1 and DQA1 (OMIM Accession ID: 142857 and 146880, respectively) and the Desmoplakin gene (DSP, OMIM Accession ID: 125647) (Table 6). HLA-DQA1 and HLA-DQB1 are likely genetic markers of the Celiac Disease [71] which was suggested to be a risk factor in CF [72, 73]. Therefore, together with our present results, we propose HLA-DQA1 and HLA-DQB1 genes, as new modifiers in CF. Desmoplakin is a cytoskeletal linker protein conferring the structural integrity of tissues by linking intermediate filament cytoskeleton from cell to cell (for review: [74]). In remodeled airways, as in CF, abnormalities of the cytokeratins and desmoplakins are observed [75]. According to the importance of intermediate filaments and cell-to-cell communications in CF [76, 77], DSP gene expression is likely involved.

Differentially expressed genes in the bibliographic search for CF and in NASH-vs-healthy re-analysis of GSE48452 were: HLA-DRA (OMIM Accession ID: 142860), GPNMB (Glycoprotein NMB, OMIM Accession ID: 604368), NCF2 (Neutrophil Cytosolic Factor 2, OMIM Accession ID: 608515), RASGRP1 (RAS Guanyl Nucleotide-Releasing Protein 1, OMIM Accession ID: 603962), LGALS3 (Lectin, Galactosidase-Binding, Soluble 3, OMIM Accession ID: 153619), PTPN13 (Protein-Tyrosine Phosphatase, Nonreceptor-Type 13; OMIM Accession ID: 600267) (Table 6).

Involvement of Class II major histocompatibility complex (MHC) genes in CF were discussed above. Because HLA Class II Polymorphism is already known to be a modifier of the pulmonary phenotype in CF [78, 79] our finding is not new.

GPNMB is a transmembrane glycoprotein expressed in the heart, lung, and small intestine [80]. The GPNMB gene is a p53- and androgen-dysregulated gene with antiproliferative and antitumorigenic effects in prostate [81]. To our knowledge, its role in CF has never been studied.

Chronic granulomatous disease (CGD) is the most common phagocyte immunodeficiency due to mutations in a gene encoding NADPH oxidase in phagocytes. As in CF, CGD is characterized by recurrent infections and inflammation in the lungs. Because patients with mutated NCF2 gene present severe infections and CGD [82, 83] NCF2 gene is a good candidate for modifying CF.

RASGRP1 plays a critical role in T cell receptor (TCR) stimulation and is essential in the Natural Killer (NK) cell activation and in the immune response [84, 85]. The NK cells are of particular importance in CF as they play a central role in clearing *P*. *aeruginosa* from the lung [86, 87].

LGALS3 gene encodes a β-galactoside-binding lectin which plays a role in apoptosis and innate immunity. Because LGALS3 corrects F508del-CFTR trafficking [88], its gene is a good candidate as a modifier in CF.

The protein encoded by PTPN13 is a tyrosine phosphatase with five PDZ (postsynaptic density protein 95/discs large/zonula occludens-1) domains that bind to plasma membrane and cytoskeleton. The C terminus of CFTR binds various PDZ domain-containing proteins including EBP50, CAL and members of the GRASP family [89]. Despite it was suggested a possible role of reduced PDZ interactions in the accelerated internalization of F508del-CFTR [90], the role of CFTR-PDZ interactions remains incompletely resolved [88]. The PTPN13 gene is therefore of interest and the encoded protein may bind and regulate CFTR.

### New potential modifier genes

In conclusion, we found three groups of potential modifier genes. From our bibliographic analysis and microarray studies, we propose here that IFI16 (NCBI ID: 3428), CCNE2 (NCBI ID: 9134) and IGFBP2 (NCBI ID: 3485) are new modifiers candidates in CF. Common genes in CF and Chronic Pancreatitis are EPHX1 (NCBI ID: 2052), HLA-DQA1 (NCBI ID: 3117) and -DQB1 (NCBI ID: 3119) and DSP (NCBI ID: 1832). Modifier genes in CF, in link to Liver Disease, are SLC33A1 (NCBI ID: 9197), GPNMB (NCBI ID: 10457), NCF2 (NCBI ID: 4688), RASGRP1 (NCBI ID: 10125), LGALS3 (NCBI ID: 3958) and PTPN13 (NCBI ID: 5783).

### Gene-pathway association of the new potential modifier genes

**Fig 6:**
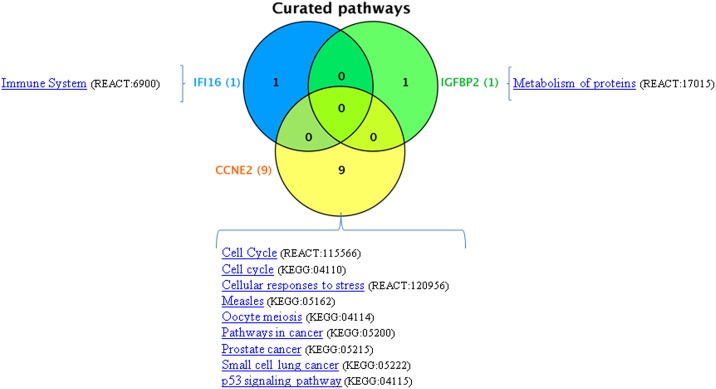
Gene-pathway association of the new potential modifier genes in KEGG and REACTOME. The 3 common genes in CF, which were retrieved from Table 6, were submitted to the pathway search, using VennViewer in CTD. 1, 1 and 9 pathways were retrieved for IFI16, IGFBP2 and CCNE2, respectively. No pathway was common between those genes.

To better understand the biological function of our new potential modifier genes, we search for their annotated pathways, which are associations established by KEGG [91] and REACTOME [92]. Biological pathways describe biological processes. Therefore, they can be used to integrate and visualize gene products in different conditions (healthy *vs* disease). KEGG and REACTOME pathway data show known molecular interactions and reaction networks. These data, integrating genes and diseases provide insights into molecular networks involving our depicted candidate genes. Indeed, pathway enrichment analysis permits to decipher biological functions associated with these genes [93]. First, the three common genes in CF (IFI16, CCNE2 and IGFBP2), which were retrieved from Table 6, were submitted to the pathway search, using VennViewer in CTD. 1, 1 and 9 pathways were retrieved for IFI16, IGFBP2 and CCNE2, respectively (Fig 6). No pathway was common between those genes. The 4 common genes in CF and Chronic Pancreatitis and the 6 common genes in CF and Liver Disease were further submitted to the pathway search (Table 7). The only common pathway between CF, Chronic Pancreatitis and Liver Disease was “Immune System” (REACTOME: 6900). The implicated genes were IFI16 for CF, HLA-DQB1 for CF plus Chronic Pancreatitis and LGALS3, NCF2 and RASGRP1 for CF plus Liver Disease (Table 7). Therefore, genes of the Immune System are likely involved in CF and in its association with Chronic Pancreatitis and Liver Disease.

**Table 7:** Associated pathways with the retrieved potential modifiers in CF, in CF and Chronic Pancreatitis and in CF and Liver Disease. Common pathways are underlined.

	Genes name	Pathway	Pathway ID
**Genes in CF (n = 3)**	CCNE2	Cell cycle	KEGG:04110
		Cell cycle	REACT:115566
		Cellular responses to stress	REACT:120956
		Measles	KEGG:05162
		Oocyte meiosis	KEGG:04114
		p53 signaling pathway	KEGG:04115
		Pathways in cancer	KEGG:05200
		Prostate cancer	KEGG:05215
		Small cell lung cancer	KEGG:05222
	IFI16I	Immune System	REACT:6900
	GFBP2	Metabolism of proteins	REACT:17015
**Genes in CF and in Chronic Pancreatitis**	DSP	Apoptosis	REACT:578
	EPHX1	Arrhythmogenic right ventricular cardiomyopathy	KEGG:05412
	HLA-DQA1	Bile secretion	KEGG:04976
		Metabolism of xenobiotics by cytochrome P450	KEGG:00980
		Allograft rejection	KEGG:05330
		Antigen processing and presentation	KEGG:04612
		Asthma	KEGG:05310
		Autoimmune thyroid disease	KEGG:05320
		Cell adhesion molecules (CAMs)	KEGG:04514
		Graft-versus-host disease	KEGG:05332
	HLA-DQB1	Immune System	REACT:6900
		Intestinal immune network for IgA	KEGG:04672
		Leishmaniasis	KEGG:05140
		Phagosome	KEGG:04145
		Rheumatoid arthritis	KEGG:05323
		Staphylococcus aureus infection	KEGG:05150
		Systemic lupus erythematosus	KEGG:05322
		Toxoplasmosis	KEGG:05145
		Tuberculosis	KEGG:05152
		Type I diabetes mellitus	KEGG:04940
		Viral myocarditis	KEGG:05416
**Genes in CF and in Liver Disease**	GPNMB	None	
	LGALS3	Immune System	REACT:6900
	NCF2	Immune System	REACT:6900
		Leishmaniasis	KEGG:05140
		Leukocyte transendothelial migration	KEGG:04670
		Osteoclast differentiation	KEGG:04380
		Phagosome	KEGG:04145
		Signal Transduction	REACT:111102
	PTPN13	None	
		Hemostasis	REACT:604
	RASGRP1	Immune System	REACT:6900
		MAPK signaling pathway	KEGG:04010
		Signal Transduction	REACT:111102
		T cell receptor signaling pathway	KEGG:04660
		Glycosphingolipid biosynthesis	KEGG:00604
		Metabolic pathways	KEGG:01100
	SLC33A1	Transmembrane transport of small molecules	REACT:15518

We further search for the diseases that are statistically enriched among our genes. Modifiers candidates in CF (IFI16, CCNE2 and IGFBP2), the common genes in CF and Chronic Pancreatitis (EPHX1, HLA-DQA1 -DQB1, DSP) and in CF in link to Liver Disease (SLC33A1, GPNMB, NCF2, RASGRP1, LGALS3 and PTPN13) were entered as a single data set in CTD. According to CTD, a disease was considered enriched if the proportion of genes annotated to it in our test set was significantly larger than the proportion of all genes annotated to it in the genome. The results are presented Table 8.

**Table 8:** Associated diseases with the retrieved potential modifiers in CF, in CF and Chronic Pancreatitis and in CF and Liver Disease. The list of genes was submitted to CTD search and associated diseases were retrieved. In bold: diseases which may present relevant involvement in CF physiopathology.

Disease Name	Disease ID	P-value	Corrected P-value (1)	Annotated Genes (input)	Genome Frequency (2)
Skin and Connective Tissue Diseases	MESH:D017437	3,24E-09	8,29E-07	DSP|EPHX1|GPNMB|HLA-DQA1|HLA-DQB1|IGFBP2|NCF2|RASGRP1	1499/41292 (3,63%)
**Female Urogenital Diseases and Pregnancy Complications**	**MESH:D005261**	**5,68E-08**	**1,45E-05**	**CCNE2|EPHX1|GPNMB|HLA-DQA1|HLA-DQB1|LGALS3|RASGRP1**	**1350/41292 (3,27%)**
Esophageal Achalasia	MESH:D004931	2,74E-07	7,03E-05	HLA-DQA1|HLA-DQB1	3/41292 (0,01%)
Skin Diseases(a)	MESH:D012871	7,73E-07	1,98E-04	DSP|EPHX1|GPNMB|HLA-DQA1|HLA-DQB1|IGFBP2	1179/41292 (2,86%)
**Female Urogenital Diseases**	**MESH:D052776**	**9,53E-07**	**2,44E-04**	**CCNE2|GPNMB|HLA-DQA1|HLA-DQB1|LGALS3|RASGRP1**	**1222/41292 (2,96%)**
Esophageal Motility Disorders	MESH:D015154	3,29E-06	8,42E-04	HLA-DQA1|HLA-DQB1	9/41292 (0,02%)
**Lung Diseases, Obstructive**(b)	**MESH:D008173**	**5,74E-06**	**0,00147**	**EPHX1|HLA-DQA1|HLA-DQB1**	**114/41292 (0,28%)**
Deglutition Disorders	MESH:D003680	7,12E-06	0,00182	HLA-DQA1|HLA-DQB1	13/41292 (0,03%)
Nephritis	MESH:D009393	7,39E-06	0,00189	HLA-DQA1|HLA-DQB1|LGALS3	124/41292 (0,30%)
Connective Tissue Diseases	MESH:D003240	1,35E-05	0,00345	HLA-DQA1|HLA-DQB1|NCF2|RASGRP1	496/41292 (1,20%)
**Immune System Diseases**	**MESH:D007154**	**1,95E-05**	**0,005**	**EPHX1|HLA-DQA1|HLA-DQB1|NCF2|RASGRP1**	**1173/41292 (2,84%)**
Autoimmune Diseases	MESH:D001327	2,08E-05	0,00533	HLA-DQA1|HLA-DQB1|NCF2|RASGRP1	554/41292 (1,34%)
Esophageal Diseases	MESH:D004935	2,15E-05	0,00549	HLA-DQA1|HLA-DQB1|LGALS3	177/41292 (0,43%)
**Male Urogenital Diseases**(c)	**MESH:D052801**	**2,61E-05**	**0,00668**	**EPHX1|GPNMB|HLA-DQA1|HLA-DQB1|LGALS3**	**1246/41292 (3,02%)**
Kidney Diseases	MESH:D007674	2,82E-05	0,00723	GPNMB|HLA-DQA1|HLA-DQB1|LGALS3	599/41292 (1,45%)
**Digestive System Diseases**(d)	**MESH:D004066**	**3,18E-05**	**0,00813**	**EPHX1|GPNMB|HLA-DQA1|HLA-DQB1|IGFBP2|LGALS3**	**2247/41292 (5,44%)**
**Celiac Disease**(e)	**MESH:D002446**	**3,44E-05**	**0,00881**	**HLA-DQA1|HLA-DQB1**	**28/41292 (0,07%)**
Pathological Conditions	MESH:D013568	3,45E-05	0,00883	DSP|EPHX1|HLA-DQA1|IGFBP2|LGALS3|SLC33A1	2280/41292 (5,52%)
Drug-Induced Liver Injury	MESH:D056486	3,62E-05	0,00927	EPHX1|HLA-DQB1|LGALS3	211/41292 (0,51

^(a)^: including Sweat Gland Diseases;

^(b)^: including Chronic Bronchitis, COPD, Mucus Inspissation of Respiratory Tract;

^(c)^: including Congenital bilateral aplasia of vas deferens;

^(d)^: including Biliary Tract Diseases, Digestive System Abnormalities, Liver Diseases and Pancreatic Diseases;

^(e)^: including Malabsorption Syndromes

^(1)^: Bonferroni-corrected p-value;

^(2)^: Genotype frequency in a population is the number of individuals with a given genotype divided by the total number of individuals in the population.

### Biological function and PPI networks association of the new potential modifier genes

For a full description of the proteins which are encoded by our candidate modifiers genes, we search for their potential interactions using the recent 10.0 version of STRING [94]. We used the basic interaction unit in STRING for functional association between proteins, derived from known experimental data. Given the importance of interactions in protein function, we search for groups of interacting proteins into functional sets, in physical complexes.

**Fig 7:**
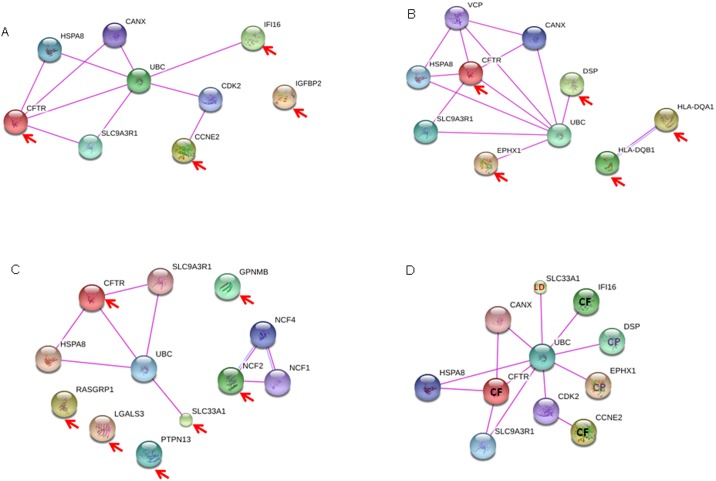
PPI networks association of the new potential modifier genes. The recent 10.0 version of STRING (High confidence ≥ 0.7) was used to search for PPI. **A.** PPI between proteins encoded by IFI16, CCNE2 and IGFBP2 (CF candidates). We observed that CFTR and IFI16 have a common partner: UBC. **B.** Interactions involving the proteins encoded by common genes in CF and *Chronic Pancreatitis* (EPHX1, HLA-DQA1 -DQB1, DSP and CFTR). EPHX1, DSP and CFTR were found to be linked by UBC. **C.** Networks formed by the proteins encoded by the genes found in CF in link to Liver Disease (SLC33A1, GPNMB, NCF2, RASGRP1, LGALS3 and PTPN13). SLC33A1 forms a protein complex together with CFTR, with UBC as an intermediate. **D.** Network formed by the proteins encoded by the genes involved in CF, in CF plus Chronic Pancreatitis and in CF plus Liver Disease, which were found in a network linked to the CFTR protein. UBC was observed as a central node. UBC is likely a key component in CF physiopathology. The proteins used in the search tool are marked by a red arrow.

We first search for high confidence (≥ 0.7) interactions involving the proteins encoded by IFI16, CCNE2 and IGFBP2 (CF candidates). We added the CFTR protein to the query. As shown in Fig 7A, a network of 9 nodes and 10 edges was obtained (clustering coefficient: 0.744). Whereas IGFBP2 did not belong to the network, we observed that CFTR and IFI16 have a common partner: Ubiquitin C (UBC, OMIM: 191340). IFI16 and UBC are involved in the innate immune signaling pathways [95]. Furthermore, Ubiquitin modification is required for the proteasomal targeting of CFTR, which is ubiquitinated in the ER during assembly and during recycling from the cell surface [96]. Therefore, modifications in the IFI16 and/or UBC gene expression could lead to modifications of the maturation of CFTR, showing the importance of IFI16 as a modifier gene in CF, in link to immunity. CCNE2 binds to CDK2 in a catalytically active complex which modulates the cell cycle progression [97] and CDK inhibition corrects F508del-CFTR proteins [98]. Together with the role of UBC upon the proteasomal targeting of CFTR, the physical and functional complexes involving CCNE2, UBC and CFTR (Fig 7A) indicates that CCNE2 is likely a modifier gene in CF.

Interactions involving the proteins encoded by common genes in CF and *Chronic Pancreatitis* (EPHX1, HLA-DQA1 -DQB1, DSP and CFTR) were searched (Fig 7B). Whereas, HLA-DQA1 and HLA-DQB1 did not belong to any complex, EPHX1, DSP and CFTR were linked by UBC. The Ubiquitin-proteasome system (UPS) is an important regulator for the intracellular trafficking of proteins and a UPS-dependent stabilization of cell-cell contacts involving DSP was shown [99]. Because a low activity of EPHX1 is a risk factor for COPD in Caucasian [63] and because an increased UPS activity is observed in COPD [100], a functional link between EPHX1 and UBC is likely involved in CF and Chronic Pancreatitis.

Fig 7C shows the networks formed by the proteins encoded by the genes found in CF in link to Liver Disease (SLC33A1, GPNMB, NCF2, RASGRP1, LGALS3 and PTPN13). GPNMB, RASGRP1, LGALS3 and PTPN13 were not found to be involved in a network, with the used search parameters. NCF2 was linked to NCF4 and to NCF1. NCF1-NCF2-NCF4 (NCF1-4, neutrophil cytosolic factors 1 to 4) from a multicomponent enzyme system (NADPH-oxidase) responsible for oxidative bursts. We failed to find any information regarding a potential involvement of the NCF1-NCF2-NCF4 enzymatic complex in CF, in PubMed. Interestingly, we observed that SLC33A1 forms a protein complex together with CFTR, with UBC as an intermediate. As mentioned above, IRE1/XBP1 controls ERAD by activating the expression of SLC33A1 [70]. UBC is also involved in ERAD which is a key element of the CFTR degradation. This reinforces our proposition that SLC33A1 is a strong modulator in CF.

Finally, a network was drawn using the proteins encoded by the genes involved in CF, in CF plus Chronic Pancreatitis and in CF plus Liver Disease, which were found in a network linked to the CFTR protein (Fig 7D). UBC was observed as a central node. It is well known that F508del-CFTR protein degradation involves Ubiquitin modification. It was previoulsy shown that the de-ubiquitinating enzyme, Ubiquitin C-terminal hydrolase-L1 (UCH-L1), is highly expressed in CF airway epithelial cells and that there is a positive correlation between UCH-L1 expression and steady state levels of Wt-CFTR protein and F508del-CFTR [101]. Although it is not sufficient to rescue F508del-CFTR, the effect of UCH-L1 upon CFTR processing shows its potential roles in CF. UBC is thus a key component in CF.

## Conclusion

CF is characterized by progressive lung disease with inflammation, pancreatic exocrine insufficiency and fatty liver disease. Liver disease is found in two-thirds of sick childrens [102–104]. Inflammation is indeed a hallmark of CF and is characterized by bacterial infection, neutrophils infiltrations in the lung and high levels of cytokines. Whereas, inflammatory response is deregulated and excessive, it is not known whether abnormal CFTR is responsible or a consequence. The most common hypothesis is that a defective CFTR leads to a decreased airway surface liquid which fails to clear infected secretions from the lung, triggering the excessive inflammatory response. It is therefore still controversial whether this hyper-inflammation is solely the result of the chronic infection or is a primary event due to CFTR defects [105]. Regarding liver, three types of Liver Disease are observed in CF patients: steatosis, cirrhosis and biliary fibrosis. Fatty infiltration of the liver being found in 30% at biopsy and in 60% at the autopsy of the CF patients, is the most common hepatic alteration [106]. In CF, PI phenotype is a hallmark in patients with two severe alleles such as F508del [16] and CF Related Diabetes (CFRD) is observed in more than 50% of patients. Whereas CFRD is a unique entity, it exhibits common features of both type 1 and 2 diabetes. However, the hallmark of CFRD is insulin deficiency, as in type 1 diabetes in which pancreatic beta cells are destroyed [107].

**Fig 8:**
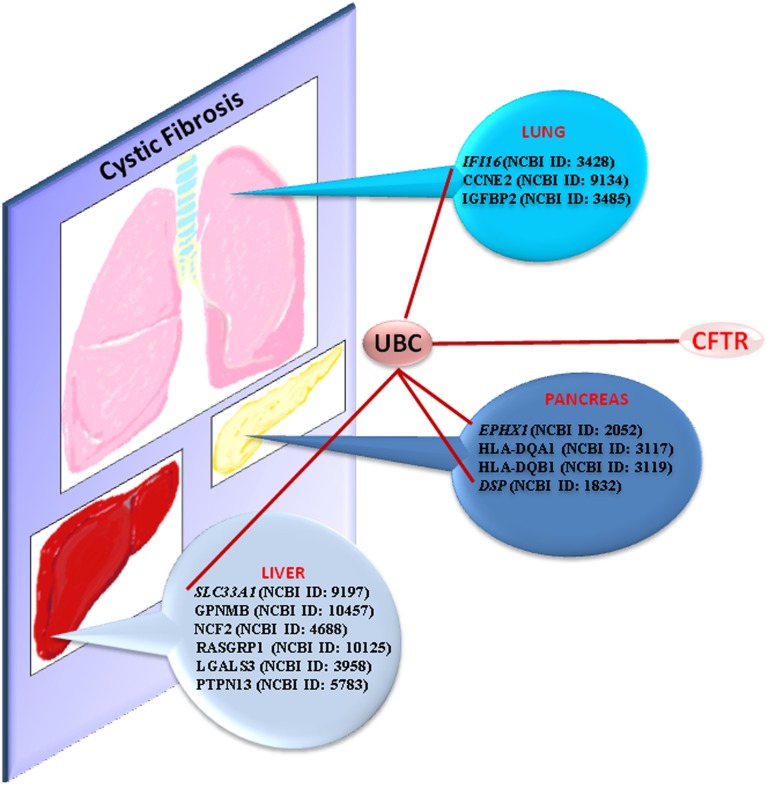
Schematic representation of the results. Candidate genes are shown in each organ. The corresponding proteins presenting PPI are linked by a red line.

Despite residual involvement of CFTR’s sequence variation upon lung function, modifiers are of main importance. Indeed, it is clear that differences in the genotype in CF are not solely responsible for the disease variability [108] and large-scale genome-wide association studies (GWAS) were undertaken to search for genetic determinants of phenotypic variation (for review, [23]). Nevertheless, further studies are still needed. Therefore, our aim was to depict new candidate genes with modifying activity in CF, in association with liver and pancreatic diseases. Using databases, bibliographic mining and gene expression analysis we found some candidate genes specific to lung, liver and pancreas. Nevertheless, two groups of different genes could be built, depending on the way they were retrieved. Unsurprisingly, databases search led to known genes involved in CF. Therefore, we focused on genes obtained by microarray results re-analysis, compared to those from our bibliographic analysis. We highlighted 3 genes specific to CF, 4 genes specific to CF and Chronic Pancreatitis and 6 genes for CF and Liver Disease. Nevertheless, we failed to find common genes in CF, Chronic Pancreatitis and Liver Disease. Pathways associated with these retrieved potential modifiers indicated that immune system is likely involved and that Ubiquitin C is the central node linking CF to liver and pancreatic disease. A schematic representation of the results is proposed in Fig 8. Despite we propose here some new genes involved in CF, we are fully aware that some other modifier genes may be missing and that our *in silico* analysis requires functional analysis to give our results a physiological relevance. Indeed, our analysis may present some limitations due to the statistical combination of results from several studies which may present variable quality and heterogeneity of the used tools. This could lead to misleading results, inherent to any systematic meta-analyses which may be non-exhaustive or over interpreted. Database and literature analysis can also be limiting because of incomplete knowledge base leading to byproduct results. Because in the manual curation of the literature we have not performed an exhaustive review of all the genes and because the used studies have been performed using different methodologies and have different power, no statistical assessment was possible here. Finally, gene expression profiles may be affected by the variability between the used cell models, their intrinsic properties and their type of statistical analysis.

In conclusion, we propose here a methodology that may be used for some other genetic disease with great variability and identify new candidate modifier genes in CF, related to lung, pancreas and liver, such as Ubiquitin C.
